# Study of the hydrogen physisorption on adsorbents based on activated carbon by means of statistical physics formalism: modeling analysis and thermodynamics investigation

**DOI:** 10.1038/s41598-020-73268-w

**Published:** 2020-09-30

**Authors:** Manel Ben Yahia, Sarra Wjihi

**Affiliations:** 1 Physics Department, Rabigh College of Science and Arts, King Abdulaziz University, Jeddah, P.O Box 344, Rabigh, 21911 Saudi Arabia; 2 Laboratory of Quantum and Statistical Physics, LR18ES18, Faculty of Science of Monastir, Monastir University, Monastir, Tunisia

**Keywords:** Chemical engineering, Physical chemistry, Surface chemistry, Biochemistry, Engineering, Materials science, Mathematics and computing, Physics

## Abstract

An advanced statistical physics model has been applied to study the hydrogen adsorption isotherm on two modified types of activated carbon**,** namely granular coal activated carbon (AC (GC)) and coconut shell activated carbon (AC (CS)). This model is established with the statistical physics approach. It is a more general model including various parameters having a defined physico-chemical sense which were discussed at different temperatures. Hence new physic-chemical interpretations of the adsorption process of hydrogen are provided. The analysis of the hydrogen uptake capacities at saturation showed that the AC (GC) adsorbent displayed a high adsorption capacity (3.21 mg/g). This due to the contribution of the number of hydrogen molecules per site (1.27) associated with the receptor sites density (0.74 mg/g) and the number of formed layers (3.42). The modeling results suggested that the hydrogen adsorption occurred by non-parallel positions on the two tested adsorbents thus evincing that the adsorption cannot be other than a multi-molecular process. The calculated adsorption energies globally varied from 7.01 to 12.92 kJ/mol, confirming the physical nature of the adsorption process for both studied systems. The thermodynamic functions, namely internal energy, enthalpy and entropy were estimated to better analyze the hydrogen sorption process. In summary, the statistical physics analysis provided reliable concrete physico-chemical interpretations of hydrogen adsorption process on carbon-based adsorbents with various microstructures to develop a storage compounds with a suitable framework for a hydrogen storage structure.

## Introduction

The growth of the world population and the technology advancement solicit an increasing energy demand which is based on a massive use of fossil fuels^1^. But their use poses two major problems. The first is a problem of availability with the continuous decrease of the global reserves of fossil fuels. The second is that the resulting greenhouse gas emissions are high and strongly contribute to global warming. It is found that hydrogen, the most abundant element in the universe, is the uncontested candidate to play a key role in the development of a new energy system, both safe and compact. However, the optimal performance of such an energy source requires the development of new storage methods. The physical storage method for hydrogen adsorption on porous structures is the best method to use for storing hydrogen in comparison with other techniques. Moreover, porous materials offer numerous advantages for the storage of hydrogen thanks to the fast hydrogen uptake and release kinetics^2–5^. One of the porous materials is activated carbon which offers good chemical stability, easy reversibility, easy availability and low price^6^. Porous carbons can be synthesized from different carbon precursors, including wood, peat, coal, coconut and fruit stones and polymers, by physical and chemical activation methods. On this basis, coals have become the principal precursor for the preparation of activated carbons due to their intrinsic microstructure and surface chemical characteristics^7,8^. To better explore and understand the physisorption mechanism of hydrogen on activated carbon adsorbents systems, the adsorption isotherms have been studied and interpreted. In this respect, various theoretical studies using adsorption models ranging from Langmuir, Langmuir–Freundlich and Tóth models have been realized^9,10^. A comparison between these models for a similar system led us to conclude that almost all of them are empirical. Hence, all their parameters are mathematical and they have no physical meaning. Their values are in relation with mathematical parameters such as a slope of the curve, its origin value, etc. None of the models used in the literature used our statistical approach, especially the particular parameters introduced into our models, such as the number of hydrogen molecules per site, the ancrage number, and the individual sorption energy. We notice that the only information deduced from some articles is the molar adsorption energy and the number of sites, sometimes synthesized empirically. The objective of this project is to provide a statistical physics framework to simulate the hydrogen adsorption isotherms of two activated carbon adsorbents [granular coal activated carbon (AC (GC)) and coconut shell activated carbon (AC (CS))]^11^. The goal of this statistical physics approach is to allow the development of many models which are able to adjust the hydrogen sorption isotherms. These models based on statistical physics have allowed an estimation of several physicochemical parameters in good relation with the sorption mechanism. The main advantage of our modeling analysis is to provide new microscopic and macroscopic information included in the sorption phenomenon that did not arise in any case of all previous works and models. In our research, we can present steric parameters like the total saturation adsorption quantities, the numbers of captured hydrogen molecules per binding site and the receptor site densities. These steric parameters could not be determined by means of classical models.

## Experimental methods

### Materials

The characterization and preparation of the two types of activated carbon (AC (GC) and AC (CS)) are discussed in^11^. The physical activation of AC (GC) and AC (CS) was performed in a vertical activation reactor placed inside an oven. The carbonization and oxidation are part of the production processes. As a first step, the granular coal and the coconut shell were heat treated by flowing O_2_ at 573 K and 100 mL/min during 6 h.

### Adsorption isotherms experiments

Hydrogen of 99.99% purity was used as adsorbate in the experiment. The experimental apparatus mainly consists of a charging cell (control volume) and a stainless steel measuring (or adsorption) cell with internal volumes of 1170 mL and 85.5 mL, respectively. The charging and the adsorption cells are connected using a small tube. The apparatus is immersed in a temperature controlled water bath with a precision of 0.2 K. The pressure of the two cells is measured using an absolute pressure transmitter (Druck, PTX 1400), with a pressure range of 0 to 40 MPa and a full scale uncertainty of 0.15%. Temperature is measured using thermocouples of class-A and type K. A thermocouple is installed in the measuring cell and is in contact with the adsorbent in the adsorption cell. This allows to obtain with precision a direct temperature of the adsorbent. The pressures and temperatures are recorded using a data logger with a sampling interval of 10 s. The detailed experimental procedures and the experimental setup used to prepare hydrogen adsorption measurement were previously described by Nasruddin et al.^11^.

## Description of the hydrogen adsorption behavior and development of the multilayer model with saturation

**Figure 1:**
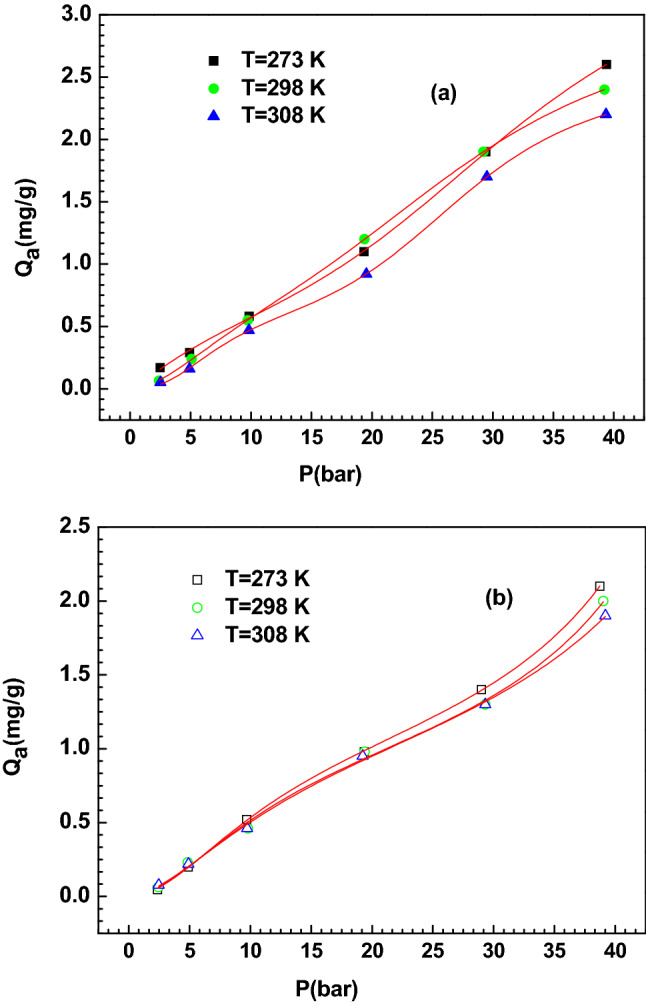
Adsorption isotherms of hydrogen on (**a**) Granular Coal Activated Carbon (AC (GC)) and (**b**) Coconut Shell Activated Carbon (AC (CS)), at different temperature and fitting by multilayer statistical physics model (MLS).

A general analysis of the experimental hydrogen adsorption data indicated that the temperature had a minor impact on the evolution of hydrogen uptake capacities (see Fig. 1) where practically the same uptake capacity was found at all the operating temperatures. It should be noted that the adsorption isotherms indicated the occurrence of an exothermic process where a slight decrease of the hydrogen adsorption capacity was shown with respect to temperature. In addition, the adsorption patterns did not reach a saturation condition at the highest investigated H_2_ partial pressure, thus hypothesizing that the adsorption could be associated to a variable number of adsorbed layers. A generalized statistical physics model was established to explore the hydrogen adsorption phenomenon and to provide a theoretical interpretation. This statistical physics model assumed that the sorption of the hydrogen molecules on the two employed adsorbents occurred via the creation of a variable number of layers, driven by two adsorption energies, which are mainly associated with these interactions: interactions between H_2_ and activated carbon based adsorbent, and interactions between H_2_ molecules (between N formed layers). It is worth noting that this model was developed by considering simple hypotheses. In a first approach, the gaseous H_2_ molecules were considered as an ideal gas, since the interaction of the hydrogen molecules is reduced to an average constant interaction represented by the chemical potential. Furthermore, the internal degrees of freedom of hydrogen are lost during sorption and only the degree of translation has been taken. This is explained by the fact that the electronic degree of freedom cannot be thermally excited. In addition, the vibrational degree of freedom can be discarded in comparison to the translational degree^12,13^. This advanced model was developed through the grand canonical ensemble in statistical physics. The expression of the grand canonical partition function of a single site is given by:

1 $${\text{z}}_{{{\text{gc}}}} = 1 + {\text{e}}^{{{\upbeta }\left( {{\upvarepsilon }_{1} + {\upmu }} \right)}} + {\text{e}}^{{{\upbeta }\left( {{\upvarepsilon }_{1} + {\upvarepsilon }_{2} + 2{\upmu }} \right)}} \frac{{1 - {\text{e}}^{{{\upbeta }\left( {{\upvarepsilon }_{2} + {\upmu }} \right)^{N} }} }}{{1 - {\text{e}}^{{{\upbeta }\left( {{\upvarepsilon }_{2} + {\upmu }} \right)}} }}$$

where (− ɛ_1_) and (− ɛ_2_) are the receptor site adsorption energy, µ is the chemical potential and β is defined as 1/k_B_T, where k_B_ is the Boltzmann constant and T is the absolute temperature.

A non-fixed number of hydrogen molecules were considered to adsorb on D_sr_ binding sites per mass unit of the AC adsorbents. The total grand canonical partition function associated with these binding sites, is supposed to be identical and independent and is given as follows:

2 $$Z_{{{\text{gc}}}} = \left[ {1 + {\text{e}}^{{{\upbeta }\left( {{\upvarepsilon }_{1} + {\upmu }} \right)}} + {\text{e}}^{{{\upbeta }\left( {{\upvarepsilon }_{1} + {\upvarepsilon }_{2} + 2{\upmu }} \right)}} \frac{{1 - {\text{e}}^{{{\upbeta }\left( {{\upvarepsilon }_{2} + {\upmu }} \right)^{N} }} }}{{1 - {\text{e}}^{{{\upbeta }\left( {{\upvarepsilon }_{2} + {\upmu }} \right)}} }}} \right]^{{D_{{sr}} }}$$

The average site occupation number can be written as^14^:

3 $${\text{N}}_{{\text{O}}} = {\text{k}}_{{\text{B}}} {\text{T}}\frac{{\partial \ln {\text{Z}}_{{{\text{gc}}}} }}{{\partial {\upmu }}} = D_{{sr}} {\text{k}}_{{\text{B}}} {\text{T}}\frac{{\partial \ln {\text{z}}_{{{\text{gc}}}} }}{{\partial {\upmu }}}$$

Generally, the adsorption of H_2_ gaseous molecules onto a binding site (S) should incorporate a stoichiometric coefficient n_ms_ as depicted in the following equation:

$$n_{{ms}} \,{\text{H}}_{2} + S \leftrightarrow S\,{\text{H}}_{{2n_{{ms}} }}$$

When the thermodynamic equilibrium is reached according to Eq. (4), the equality of the different chemical potentials can be given by: µ_m_ = µ/n_ms_, where µ is the chemical potential of the sorbed molecules, n_ms_ the number of hydrogen molecules per binding site and µ_m_ is the chemical potential of the hydrogen gaseous molecules. By utilizing the ideal gas approximation, the chemical potential in the gaseous state can be given by the following equation^15^:

$${\upmu }_{{\text{m}}} = {\text{k}}_{{\text{B}}} \,{\text{T}}\ln\frac{{\text{N}}}{{{\text{Z}}_{{\text{g}}} }}$$

where ${\text{Z}}_{{\text{g}}} = {\text{Z}}_{{{\text{gtr}}}}$ is the translation partition function of the hydrogen gaseous molecules which can be written as^15,16^:

$${\text{Z}}_{{\text{g}}} = {\text{Z}}_{{{\text{gtr}}}} = {\text{V}}\left( {\frac{{2{\uppi }\,{\text{mk}}_{{\text{B}}} {\text{T}}}}{{{\text{h}}^{2} }}} \right)^{{3/2}}$$

where *h, V*, and *m* are the Planck constant, the volume of the hydrogen gas and the sorbed molecule mass, respectively. The translation partition function per unit of volume z_gtr_, for an ideal gas, can be represented as a function of the hydrogen saturated vapor pressure P_vs_ and the hydrogen vaporization energy ΔE^v^ by the equation^14–19^:

7 $$Z_{{{\text{gtr}}}} = {\upbeta }\,{\text{P}}_{{{\text{vs}}}} {\text{e}}^{{\frac{{{\Delta }{\text{E}}^{{\text{v}}} }}{{{\text{RT}}}}}}$$

where

8 $${\text{P}}_{{{\text{vs}}}} = \left( {\frac{{\left( {2{\uppi }\,{\text{mk}}_{{\text{B}}} {\text{T}}} \right)^{{3/2}} }}{{{\text{h}}^{3} }}} \right)\,\,\,\left( {{\text{k}}_{{\text{B}}} \,{\text{T}}} \right)\,{\text{e}}^{{ - \frac{{{\Delta }{\text{E}}^{{\text{v}}} }}{{{\text{RT}}}}}}$$

Using Eq. (4) and the number of average site occupation N_o_, the average number of sorbed molecules is written as:

$${\text{Q}}_{{\text{a}}} = {\text{n}}_{{ms}} \,{\text{N}}_{{\text{o}}}$$

The adsorbed quantity is written as:

10 $${\rm Q}_{{\rm a}} = {\rm n}_{{\rm ms}} \cdot {\rm D}_{sr} \frac{{ - \frac{{2\left( {\frac{{\rm P}}{{{\rm P}_{{\rm hs}1} }}} \right)^{{2{\rm n}_{{\rm ms}} }} }}{{\left( {1 - \left( {\frac{{\rm P}}{{{\rm P}_{{\rm hs}1} }}} \right)^{{{\rm n}_{{\rm ms}} }} } \right)}} + \frac{{\left( {\frac{{\rm P}}{{{\rm P}_{{\rm hs}1} }}} \right)^{n} \left( {1 - \left({\frac{{\rm P}}{{{\rm P}_{{\rm hs}1} }}} \right)^{{2{\rm n}_{{\rm ms}} }} } \right)}}{{\left( {1 - \left( {\frac{{\rm P}}{{{\rm P}_{{\rm hs}1} }}} \right)^{{{\rm n}_{{\rm ms}} }} } \right)^{2} }} + \frac{{2\left( {\frac{{\rm P}}{{{\rm P}_{{\rm hs}1} }}} \right)^{{{\rm n}_{{\rm ms}} }} \left( {\frac{{\rm P}}{{{\rm P}_{{\rm hs}2} }}} \right)^{{{\rm n}_{{\rm ms}} }} \left( {1 - \left( {\frac{{\rm P}}{{{\rm P}_{{\rm hs}2} }}} \right)^{{({\rm n}_{{\rm ms}} {\rm N})}} } \right)}}{{\left( {1 - \left( {\frac{{\rm P}}{{{\rm P}_{{\rm hs}2} }}} \right)^{{{\rm n}_{{\rm ms}} }} } \right)}} + - \frac{{\left( {\frac{{\rm P}}{{{\rm P}_{{\rm hs}1} }}} \right)^{{{\rm n}_{{\rm ms}} }} \left( {\frac{{\rm P}}{{{\rm P}_{{\rm hs}2} }}} \right)^{{{\rm n}_{{\rm ms}} }} \left( {\frac{{\rm P}}{{{\rm P}_{{\rm hs}2} }}} \right)^{{\left( {{\rm n}_{{\rm ms}} {\rm N}} \right)}} {\rm N}}}{{\left( {1 - \left( {\frac{{\rm P}}{{{\rm P}_{{\rm hs}2} }}} \right)^{{{\rm n}_{{\rm ms}} }} } \right)}} + \frac{{\left( {\frac{{\rm P}}{{{\rm P}_{{\rm hs}1} }}} \right)^{{{\rm n}_{{\rm ms}} }} \left( {\frac{{\rm P}}{{{\rm P}_{{\rm hs}2} }}} \right)^{{\left( {2{\rm n}_{{\rm ms}} } \right)}} \left( {1 - \left( {\frac{{\rm P}}{{{\rm P}_{{\rm hs}2} }}} \right)^{{({\rm n}_{{\rm ms}} {\rm N})}} } \right)}}{{\left( {1 - \left( {\frac{{\rm P}}{{{\rm P}_{{\rm hs}2} }}} \right)^{{{\rm n}_{{\rm ms}} }} } \right)^{2} }}}}{{\frac{{\left( {1 - \left( {\frac{{\rm P}}{{{\rm P}_{{\rm hs}1} }}} \right)^{{2{\rm n}_{{\rm ms}} }} } \right)}}{{\left( {1 - \left( {\frac{{\rm P}}{{{\rm P}_{{\rm hs}1} }}} \right)^{{{\rm n}_{{\rm ms}} }} } \right)}} + \frac{{\left( {\frac{{\rm P}}{{{\rm P}_{{\rm hs}1} }}} \right)^{{{\rm n}_{{\rm ms}} }} \left( {\frac{{\rm P}}{{{\rm P}_{{\rm hs}2} }}} \right)^{{{\rm n}_{{\rm ms}} }} \left( {1 - \left( {\frac{{\rm P}}{{{\rm P}_{{\rm hs}2} }}} \right)^{{\left( {{\rm n}_{{\rm ms}} {\rm N}} \right)}} } \right)}}{{\left( {1 - \left( {\frac{{\rm P}}{{{\rm P}_{{\rm hs}2} }}} \right)^{{{\rm n}_{{\rm ms}} }} } \right)}}}}$$

The theoretical equation of this multilayer model presents numerous physicochemical parameters that can be determined from the experimental data of hydrogen adsorption on the two ACs by modeling examination: the number of hydrogen molecules per binding site n_ms_, the density of binding sites D_sr_, the number of sorbed layers N and the pressures at half saturation P_hs1_ and P_hs2_ for the first and the Nth layers respectively.

## Results and discussion

### Simulation: Levenberg–Marquardt iterating algorithm

A computer simulation was used to adjust the hydrogen adsorption isotherm with the proposed models. All the hydrogen sorption isotherms were adjusted by three statistical physics models; multilayer model with saturation (MLS), double layer model with two energies (DLT) and single layer model with one energy (MLO) using microcal origin software (Origin Lab, Northampton, MA). In Table 1, the partition functions of the three proposed models are shown. In this paper, two adjusting parameters were employed: the determination coefficient R^2^ and the residual root mean square error (RMSE). It should be noted that a sorption isotherm is in a good correlation with the tested statistical physics model, when the coefficient of adjustment R^2^ tends to the unit and the values of residual root mean square RMSE are close to zero. All the values of the determination coefficient (R^2^) and RSME of all the established models, as adjusted to the adsorption isotherm of hydrogen on AC (GC) and AC (CS), are summarized in Table 2. As can be observed, the multilayer adsorption model presents the perfect adjusting results since the values of RMSE are close to zero (from 0.009 to 0.012) and the values of R^2^ are close to unity (0.9982–0.9995) in comparison with the other applied models. Consequently, it was adopted for the analysis and discussion of the hydrogen adsorption process. The fitting of the adsorption isotherms of hydrogen on the two ACs using the multilayer model with saturation is illustrated in Fig. 1. We also present in Table 3 the adjusted values of the parameters of the selected model i.e. the hydrogen molecules per binding site n_ms_, the receptor sites density D_sr_, the hydrogen uptake capacity at saturation Q_asat_ and the estimated values of adsorption energies − ε_1_ and − ε_2_.

**Table 1:** Partition functions of the proposed models.

Name of tested model	Partition function	Reference
Monolayer with one energy (MLO)	$Z_{{{\text{gc}}}} = \left( {1 + {\text{e}}^{{{\upbeta }\left( {{\upvarepsilon }_{1} + {\upmu }} \right)}} } \right)^{{D_{{sr}} }}$	^15,16^
Double layer with two energies (DLT)	$Z_{{{\text{gc}}}} = \left( {1 + {\text{e}}^{{{\upbeta }\left( {{\upvarepsilon }_{1} + {\upmu }} \right)}} + {\text{e}}^{{{\upbeta }\left( {{\upvarepsilon }_{1} + {\upvarepsilon }_{2} + 2{\upmu }} \right)}} } \right)^{{D_{{sr}} }}$	^15,16^
Multilayer with saturation (MLS)	$Z_{{{\text{gc}}}} = \left( {1 + {\text{e}}^{{{\upbeta }\left( {{\upvarepsilon }_{1} + {\upmu }} \right)}} + {\text{e}}^{{{\upbeta }\left( {{\upvarepsilon }_{1} + {\upvarepsilon }_{2} + 2{\upmu }} \right)}} \frac{{1 - {\text{e}}^{{{\upbeta }\left( {{\upvarepsilon }_{2} + {\upmu }} \right)^{N} }} }}{{1 - {\text{e}}^{{{\upbeta }\left( {{\upvarepsilon }_{2} + {\upmu }} \right)}} }}} \right)^{{D_{{sr}} }}$

**Table 2:** Values of coefficient of determination R^2^ and RMSE of each model.

Adsorbent	T (K)	Model		
		MLO	DLT	MLS
**R**^**2**^				
AC (GC)	273	0.9721	0.9893	0.9993
	298	0.9781	0.9831	0.9995
	308	0.9657	0.9842	0.9982
**RMSE**				
AC (CS)	273	0.371	0.105	0.012
	298	0.353	0.145	0.035
	308	0.497	0.124	0.009

**Table 3:** Estimated values of parameters of MLS model for the adsorption of hydrogen on AC (GC) and AC (CS).

Adsorbent	Temperature (K)	Parameters					
		n_ms_	D_sr_(mg/g)	N_T_	Q_asat_ (mg/g)	− ε_1_ (kJ/mol)	− ε_2_ (kJ/mol)
AC (GC)	273	1.27	0.74	3.42	3.21	− 10.25	− 7.44
	298	1.86	0.43	3.76	3.00	− 11.88	− 8.89
	308	2.62	0.23	4.13	2.48	− 12.92	− 9.47
AC (CS)	273	1.15	0.64	2.87	2.11	− 10.02	− 7.01
	298	1.66	0.38	3.24	2.04	− 11.52	− 8.45
	308	2.43	0.21	3.75	1.91	− 12.49	− 9.21

### Interpretation of the parameters of statistical physics models

The adsorption of hydrogen on AC (GC) and AC (CS) at three operating temperatures was interpreted and analyzed via the adjusted values of the different parameters in terms of steric and energetic points of view.

#### Steric analysis

##### The hydrogen adsorption capacity at saturation (Q_asat_)

The adopted statistical physics model was able to determine the values of the uptake capacity at saturation at different temperatures and to provide a clarification of the described profile of the hydrogen adsorption isotherms. We note that the expression of this parameter is obtained by the correlation of the three parameters of the saturated multilayer model: Q_asat_ = n_ms_. D_sr_ (1 + N). Table 3 shows that the trend of this parameter satisfies the following ranking: Q_asat_ (H_2_-AC (GC)) > Q_asat_ (H_2_-AC (CS)). This result indicates that the AC (GC) exhibits the highest adsorption capacity of hydrogen molecules compared to the AC (CS) adsorbent.

**Figure 2:**
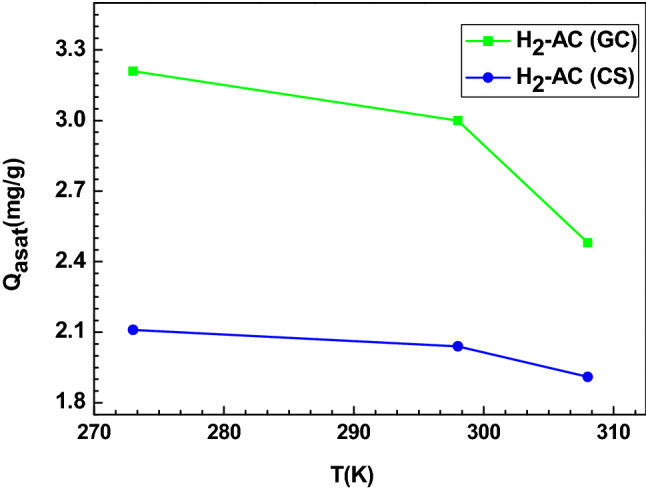
Effect of temperature on the parameter Q_asat_ for the adsorption of hydrogen on AC (GC) and AC (CS) adsorption system.

This trend is mainly due to the specific beneficial characteristics of the AC (GC) adsorbent, such as well-developed internal pore structure, high surface area and high affinity of the binding sites of the AC (GC) surface^6–8^. In fact, according to the characterization data^11^, the AC (GC) has the largest surface area (729.944 m^2^/g) in comparison with AC (CS) adsorbent (414.900 m^2^/g). It is clear that this texture parameter is the main feature affecting the hydrogen adsorption amounts. Figure 2 reports the hydrogen adsorption capacities at saturation with respect to temperature for the two adsorbents. It can be observed that AC (GC) and AC (CS) presented a similar behavior in the slope of the curve, with a gradual decrease in the uptake capacity as a function of the different tested operating temperatures. This can be explained by the fact that the increase of temperature could improve the thermal collisions between the hydrogen molecules; therefore, the hydrogen adsorption binding on both ACs was decreased, which was related to an exothermic sorption mechanism.

##### The number of hydrogen molecules per site (n_ms_)

**Figure 3:**
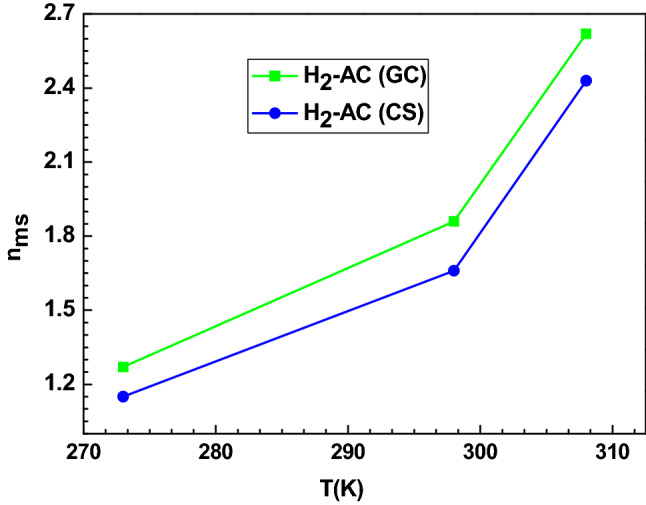
Effect of temperature on the parameter n_ms_ for the adsorption of hydrogen on AC (GC) and AC (CS) adsorption system.

The number of captured molecules per site plays a significant role to elucidate the hydrogen adsorption mechanism, thus the coefficient n_ms_ is able to outline the adsorption orientation at different temperatures. In addition, it can determine the aggregation degree of hydrogen molecules. To describe the adsorption position, it is sufficient to compare the n_ms_ adjusted values by the unity. Two cases can be examined: n_ms_ values lower than unity, i.e. each binding site of the AC adsorbents can receive a part of the hydrogen molecules. Therefore, the adsorption position is parallel. In the second case, the n_ms_ values are higher or equal to unity; each binding site can receive one or more hydrogen molecules. Table 3 shows that all the calculated values of this parameter were higher than unity at different operating temperature. Overall, the values of n_ms_ varied from 1.27 to 2.89 and from 1.45 to 2.43 for the adsorption systems H_2_-AC(GC) and H_2_-AC(CS) , respectively. This result demonstrated a non-parallel position of hydrogen on both AC adsorbents involving a multi-molecular process, thereby the hydrogen molecules could link to a single adsorption site. As stated, the aggregation degree of the hydrogen molecules can also be determined and interpreted by the parameter n_ms_. The results showed that this aggregation process could happen practically at three tested temperatures where n_ms_ ≈ 1 for the monomer, n_ms_ ≈ 2 for the dimmer and n_ms_ ≈ 3 for the trimmer for both AC adsorbents. We can deduce that a low aggregation degree indicates that the hydrogen adsorption system is not highly thermally activated. In other words, the temperature has a minor impact on the aggregation process. A comparison between the two adsorption systems indicated that this parameter varied as follows: n_ms_ (H_2_-AC (GC)) > n_ms_ (H_2_-AC (CS)). This behavior could be mainly explained by the existence of a variety of functional groups on the surfaces of the AC (GC) adsorbents, which could expedite and facilitate the binding of the hydrogen molecules via specific interactions with the lead functional groups responsible of the hydrogen adsorption. In this respect, more molecules per site were captured by the AC (GC**)** adsorbent, affirming by means of statistical physics, that this adsorbent is more adequate for hydrogen storage. From Fig. 3, we notice that the number of captured hydrogen molecules per binding site is slightly increased as a function of temperature for both AC adsorbents. This behavior is due to the thermal agitation.

##### The receptor site density (D_sr_)

**Figure 4:**
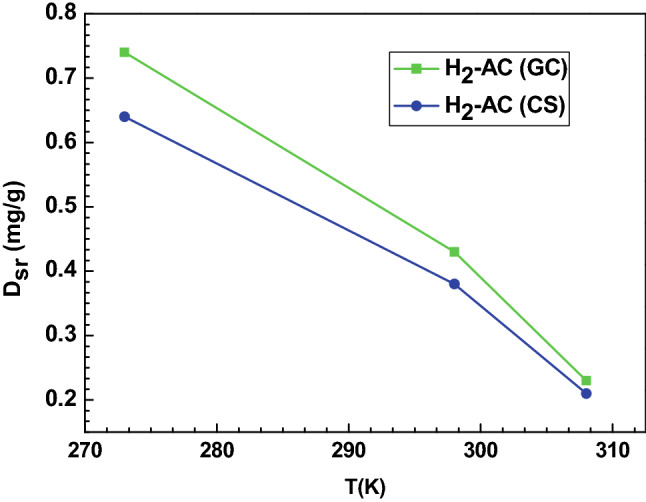
Effect of temperature on the parameter D_sr_ for the adsorption of hydrogen on AC (GC) and AC (CS) adsorption system.

D_sr_ is a useful parameter which provides indication on the density distribution of receptor sites available to the hydrogen molecules. It expresses the capacity of the AC adsorbents’ surface to capture the hydrogen molecules. The evolution of the density of the receptor sites with respect to temperature is illustrated in Fig. 4. It can be observed that this parameter varies in an opposite manner with the temperature. The opposite trend of D_sr_ expresses an antagonistic effect. Indeed, both n_ms_ and D_sr_ give an indication about the hydrogen adsorption amount on the AC adsorbents and they are not independent of each other. When the number of hydrogen molecules rises, this creates a steric hindrance for the filling of the corresponding D_sr_ spots, and therefore D_sr_ tends to decrease. This evidence can be explained by thermal agitations, which characterize a normal adsorption mechanism described by the negative binding energy and the exothermicity of the mechanism, due to the adsorption energy affected by the thermal collisions.

##### The total number of formed layers (N_T_ = 1 + N)

**Figure 5:**
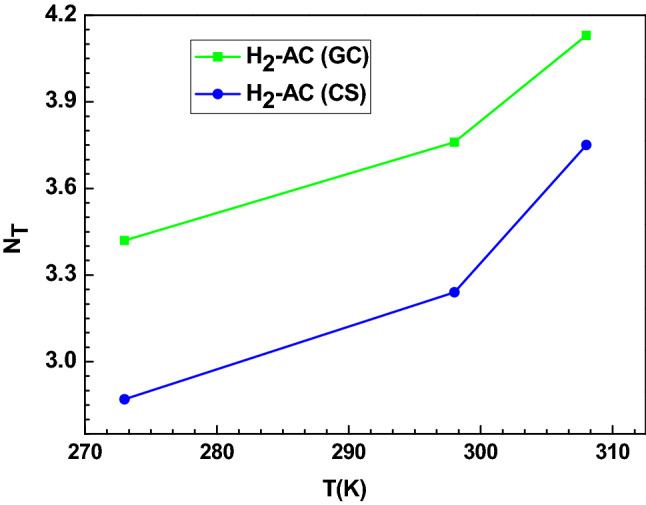
Effect of temperature on the parameter N_T_ for the adsorption of hydrogen on AC (GC) and AC (CS) adsorption system.

The physicochemical parameter N_T_ is the global number of hydrogen layers adsorbed on both AC adsorbents and its calculated values are given in Table 3 and illustrated in Fig. 5. It should be noted that the created layers varied from 3.42 to 4.13 and from 2.87 to 3.75 for systems H_2_-AC (GC) and H_2_-AC (CS), respectively. This theoretical evidence indicates that the adsorption of hydrogen on the AC (GC) is practically done by the creation of four layers and by the creation of three or almost four hydrogen layers for the AC (CS) system, rising with the operating temperature. For instance, at T = 308 K, the N_T_ value is equal to 3.75. This value indicates that three or four layers are constituted of hydrogen molecules on AC (CS). It can be analyzed by the respective proportions (z) and (1 − z) or numerically by 25% of the hydrogen molecules created three layers and 75% of the hydrogen molecules constituted four layers. Hence, the four layers are not totally occupied. According to the behavior of N_T_ as a function of temperature (Fig. 5), it can be deduced that the normal thermal agitation relatively improved the creation of layers for the hydrogen adsorption on both ACs.

#### Energetic analysis: study of the adsorption energies

An energetic study is significant to describe the interactions between the hydrogen molecules and the hydrogen molecules with the AC adsorbents. Referring to the expression of the MLS model, two pressures at half saturation P_hs1_ and P_hs2_ are associated to the binding energies of various sorbed layers. From these two parameters (P_hs1_ and P_hs2_), we can estimate the values of the two adsorption energies and hence interpret their variation with respect to temperature. The formulas of these energies are written as:

11 $$- \varepsilon _{1} = - k_{B} \,{\text{T}}\ln\frac{{{\text{P}}_{{{\text{vs}}}} }}{{{\text{P}}_{{{\text{hs}}1}} }}$$

12 $$- \varepsilon _{2} = - k_{B} \,{\text{T}}\,\ln\frac{{{\text{P}}_{{{\text{vs}}}} }}{{{\text{P}}_{{{\text{hs}}2}} }}$$

where k_B_ is the Boltzmann constant and P_vs_ is the saturated vapor pressure of hydrogen at the corresponding temperature.

**Figure 6:**
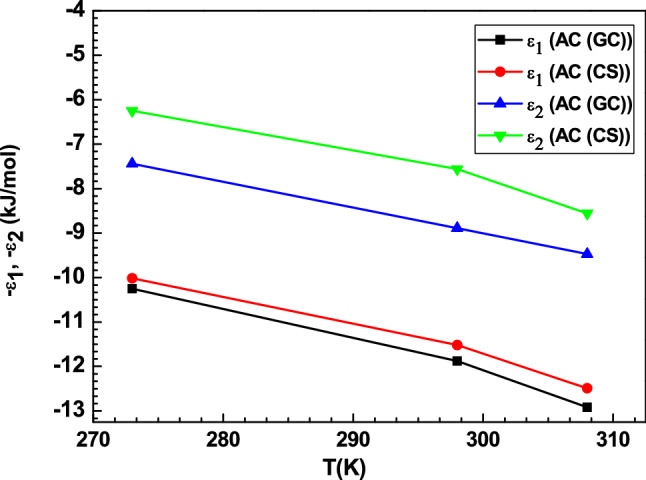
Effect of temperature on the hydrogen adsorption energy (− ε_1_ and − ε_2_) of AC (GC) and AC (CS) adsorption system.

Figure 6 illustrates the evolution of the adsorption energies − ε_1_ and − ε_2_ with respect to temperature. The adsorption energies values estimated in this paper are about 6–12 kJ/mol for the two AC adsorbents (Table 3) and are comparable to many other works^9,20–23^. These values indicate that binding between hydrogen and the AC surface takes place via a physical adsorption. According to Fig. 6, it can be observed that the adsorption energy decreases when the temperature increases, which was associated to the reduction of the uptake capacity in agreement with the exothermic nature of the hydrogen adsorption process.

### Evaluation of the thermodynamic functions

The thermodynamic functions can be estimated using a general analysis of the hydrogen adsorption system with the theoretical formula of the multilayer statistical physics model. The entropy, the Gibbs free energy and the internal energy were estimated and interpreted in the following subsections.

#### Entropy

The study of the entropy has a significant interest because it provides details about the order and disorder of the hydrogen molecules on the AC adsorbent surface. The analytical equation of the adsorption entropy can be derived using the grand potential and the selected grand canonical partition function as follows^24^:

13 $${\text{J}} = - {\text{k}}_{{\text{B}}} {\text{T}}\ln {\text{Z}}_{{{\text{gc}}}}$$

14 $${\text{J}} = - \frac{{\partial \ln {\text{Z}}_{{{\text{gc}}}} }}{{\partial {\upbeta }}} - {\text{TS}}_{{\text{a}}}$$

By equalizing these two expressions, we obtain the following equation:

15 $$\frac{{{\text{S}}_{{\text{a}}} }}{{{\text{k}}_{{\text{B}}} }} = - {\upbeta }\frac{{\partial \ln\,Z_{{{\text{gc}}}} }}{{\partial {\upbeta }}} + \ln {\text{Z}}_{{{\text{gc}}}}$$

**Figure 7:**
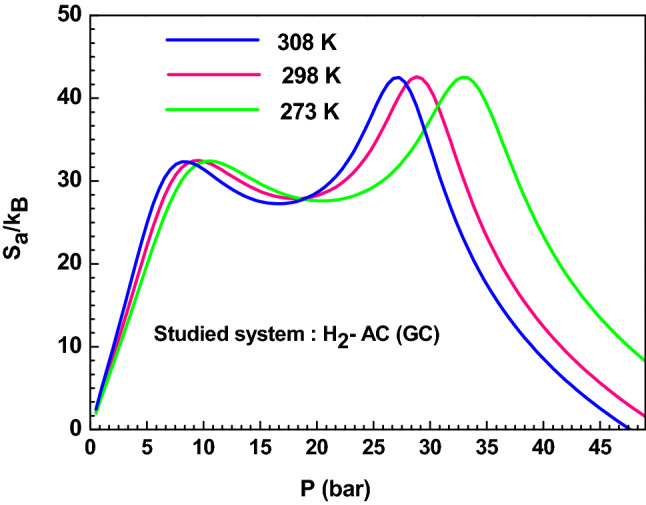
Variation of the entropy versus pressure for the AC (GC) adsorption system at different temperature.

The entropy variation as a function of the hydrogen pressure is illustrated in Fig. 7. From this figure, it can be globally observed that the entropy presents two different trends below and above the half saturation pressure values (P_hs1_) and (P_hs2_). In fact, the entropy curves rise with the pressure before the half-saturation and decrease after this point. When the adsorption pressure is lower than P_hs1_, the hydrogen molecules have a high probability to select a binding site to adsorb and hence the disorder rises at the AC (GC) surface with the pressure. After the half-saturation, the hydrogen molecules have few possibilities to select the adsorbent site since the AC (GC) surface tends to the saturation and hence tends towards the order. The entropy can reach zero when the saturation is attained.

#### Gibbs free energy

The Gibbs free energy characterizes the spontaneity of the adsorbed systems. The behavior of the external environment was integrated and estimated by the following expression^24^:

16 $${\text{G}} = {\upmu }\,{\text{Q}}_{{\text{a}}}$$

where Q_a_ is the sorbed quantity and μ is the chemical potential of the receptor site.

**Figure 8:**
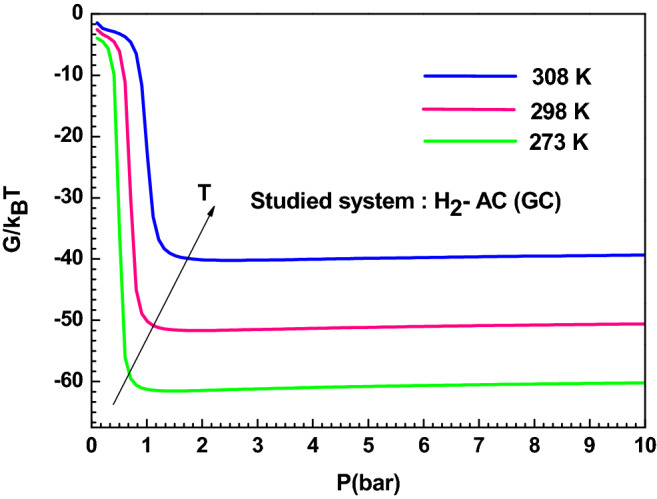
Variation of the free enthalpy versus pressure for the AC (GC) adsorption system at different temperature.

The estimated values of the Gibbs free energy, illustrated in Fig. 8, are negative, demonstrating that the sorption process occurred spontaneously. In addition, the Gibbs free energy values decreased in module with the increasing temperature for AC (GC) adsorbent, which demonstrates a reduction in adsorption feasibility at high temperatures. The temperatures prevent the hydrogen adsorption mechanism from occurring.

#### Internal energy

The internal energy is estimated using the following expressions^24^:

17 $${\text{E}}_{{{\text{int}}}} = \sum {{\upvarepsilon }_{{\text{i}}} } \,{\text{N}}_{{\text{i}}}$$

18 $${\text{E}}_{{{\text{int}}}} = - \frac{{\partial \ln {\text{Z}}_{{{\text{gc}}}} }}{{\partial {\upbeta }}} + \frac{\mu }{{\upbeta }}\left( {\frac{{\partial \ln {\text{Z}}_{{{\text{gc}}}} }}{{\partial \mu }}} \right)$$

**Figure 9:**
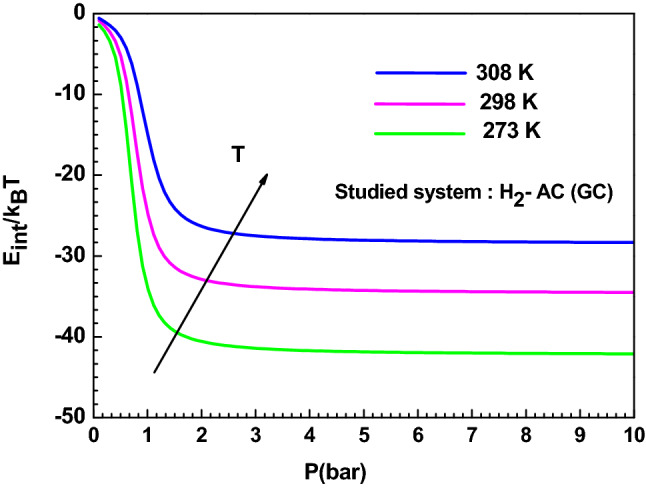
Variation of the internal energy versus pressure for the AC (GC) adsorption system at different temperature.

The variation of this potential function is depicted in Fig. 9. It can be noticed that the E_int_ values are negative, affirming that the AC (GC) hydrogen adsorption system evolve spontaneously because they release energy. Also, it can be observed that E_int_ increases algebraically but decreases in module when the temperature rises, and this is mainly due to the thermal agitation.

## Conclusion

The established multilayer model with saturation reproduces much more accurately the experimental equilibrium data, compared to the tested models. The adsorption isotherms of hydrogen on the two ACs based adsorbents were explained and discussed in light of this statistical physics model, at three operating temperatures. The analysis of the number of hydrogen molecules per binding site for the H_2_-AC(GC) and H_2_-AC(CS) systems indicates that the hydrogen molecules could be aggregated in a monomer (n_ms_ = 1) and a dimmer (n_ms_ = 2) form on the receptor sites. The hydrogen aggregation degree rose with the increasing temperature, demonstrating that the two ACs adsorbents were energetically activated. The increase of temperature has conducted to the reduction in the density of the receptor sites for both tested adsorbents due to thermal agitation. The study of the adsorbed amount at saturation indicates that the quite temperature is the perfect condition to get a good storage of hydrogen on the AC adsorbents. The estimated values of the adsorption energies indicate that hydrogen is physisorbed. To describe the sorption mechanism, the entropy was estimated according to the selected multilayer model. The variation of the internal energy and the Gibbs free energy indicates that the hydrogen adsorption on both AC adsorbents is spontaneous till the saturation is reached.
